# Self-assessment of cochlear health by cochlear implant recipients

**DOI:** 10.3389/fneur.2022.1042408

**Published:** 2022-11-16

**Authors:** Faizah Mushtaq, Andrew Soulby, Patrick Boyle, Terry Nunn, Douglas E. H. Hartley

**Affiliations:** ^1^National Institute for Health Research (NIHR) Nottingham Biomedical Research Centre (BRC), Nottingham, United Kingdom; ^2^Hearing Sciences, Division of Clinical Neuroscience, School of Medicine, University of Nottingham, Nottingham, United Kingdom; ^3^Rinri Therapeutics Ltd., Innovation Centre, Sheffield, United Kingdom; ^4^St. Thomas' Hearing Implant Centre, St. Thomas' Hospital, London, United Kingdom; ^5^Advanced Bionics GmbH, European Research Center, Hannover, Germany; ^6^Nottingham University Hospitals National Health Service (NHS) Trust, Queen's Medical Centre, Nottingham, United Kingdom

**Keywords:** cochlear implant, hearing loss, electrode impedance, electrocochleography, electrically-evoked compound action potential, neural response telemetry

## Abstract

Recent technological advances in cochlear implant (CI) telemetry have enabled, for the first time, CI users to perform cochlear health (CH) measurements through self-assessment for prolonged periods of time. This is important to better understand the influence of CH on CI outcomes, and to assess the safety and efficacy of future novel treatments for deafness that will be administered as adjunctive therapies to cochlear implantation. We evaluated the feasibility of using a CI to assess CH and examined patterns of electrode impedances, electrically-evoked compound action potentials (eCAPs) and electrocochleography (ECochGs), over time, in a group of adult CI recipients. Fifteen subjects were trained to use the Active Insertion Monitoring tablet by Advanced Bionics, at home for 12 weeks to independently record impedances twice daily, eCAPs once weekly and ECochGs daily in the first week, and weekly thereafter. Participants also completed behavioral hearing and speech assessments. Group level measurement compliance was 98.9% for impedances, 100% for eCAPs and 99.6% for ECochGs. Electrode impedances remained stable over time, with only minimal variation observed. Morning impedances were significantly higher than evening measurements, and impedances increased toward the base of the cochlea. eCAP thresholds were also highly repeatable, with all subjects showing 100% measurement consistency at, at least one electrode. Just over half of all subjects showed consistently absent thresholds at one or more electrodes, potentially suggesting the existence of cochlear dead regions. All subjects met UK NICE guidelines for cochlear implantation, so were expected to have little residual hearing. ECochG thresholds were, unsurprisingly, highly erratic and did not correlate with audiometric thresholds, though lower ECochG thresholds showed more repeatability over time than higher thresholds. We conclude that it is feasible for CI users to independently record CH measurements using their CI, and electrode impedances and eCAPs are promising measurements for objectively assessing CH.

## Introduction

Hearing loss is principally caused by sensory hair cell death or dysfunction, and, subsequently, auditory neuron degeneration (1). Cochlear implants (CIs) are often considered the gold standard treatment for severe hearing loss and although most recipients receive benefit, outcomes vary widely (2–4). Cochlear health (CM) can be broadly defined as a cochlea free from disease, illness or injury as evidenced by good hair cell and spiral ganglion function, aligned with a lack of evidence of inflammation. Consequently, variations in CM could account for some of the variability observed in CI outcomes (5, 6). Indeed, there is a worldwide effort to develop novel biological treatments to address the deficiencies of hearing devices, such as pharmacological treatments, and stem cell and gene-based approaches (7, 8). These aim to restore natural hearing by repairing or replacing damaged cells within the inner ear. The privileged location of the inner ear creates a challenge for the delivery of such treatments and for their safety and efficacy assessments. Therefore, it is anticipated that many early-phase human trials will involve delivery of novel therapeutics as an adjunct to cochlear implantation. Until fairly recently, it has been notoriously difficult to measure CH. However, due to technological advances, an additional advantage of an adjunctive approach is that post-operative monitoring of CH can be performed telemetrically using the CI electrode (9). Unlike other assessment methods, the CI electrode provides direct access to the cochlea, enabling CH parameters to be continuously recorded.

A number of established biomarkers can be used to evaluate CH, including electrode impedances, electrically-evoked compound action potentials (eCAPs) and electrocochleography (ECochGs) (9–11). Electrode impedances are used to evaluate the interface between the intra-cochlear electrode array and the tissue surrounding it, and are sensitive to inflammatory changes within the cochlea (6, 12, 13). Whilst a direct comparison between electrode impedances and the intra-cochlear inflammatory response in human listeners is difficult, animal models have shown that inflammatory tissue growth around an electrode array in guinea pigs is positively correlated with intra-cochlear electrode impedances measurements (10). Electrode impedances have been shown to stabilize after the first few weeks and months post-implantation in functioning electrodes (14, 15).

The eCAP is a direct measurement of a synchronized neural response generated by auditory nerve fibers that makes it feasible to evaluate the health status of the auditory nerve (9, 16). It is sensitive to electrode impedances, electrode placement and the health status of auditory nerve fibers near the recording electrode (17, 18). Recent literature has focused on using the eCAP to evaluate neural survival (19–21). Although a direct comparison between eCAP responses and spiral ganglion cell density in human listeners is not feasible, animal studies have shown that spiral-ganglion nerve survival in guinea pigs is positively correlated with eCAP responses (22, 23).

An EcochG enables the non-invasive monitoring of residual acoustic auditory function, including hair cell responses, that strongly correlates with audiometric thresholds (11). Importantly, our study involved adults who fit the UK National Institute for Health and Care Excellence (NICE) criteria for cochlear implantation (24), so we expected to record few EcochG responses. The EcochG was included in the test paradigm due to the exploratory nature of this study.

Together, electrode impedances, eCAPs and ECochGs provide a multi-faceted snapshot of the health status of the cochlea. To date, these measurements have not been assessed repeatedly over time due to the reliance on patients attending clinics, making regular, long-term measurements impractical. However, recent technological advances now enable participants to take recordings themselves (25). Therefore, we sought to (i) evaluate the feasibility of using a CI to measure CH through participant self-assessment and (ii) examine the pattern of CH measurements over time. We hypothesized that our group of adult CI recipients would show stable impedances and eCAPs over time, indicating no change in the intra-cochlear inflammatory response and sensory nerve survival, respectively. We also hypothesized that (i) ECochGs would only be recorded in subjects with residual hearing, measured using Pure Tone Audiometry (PTA), (ii) that lower ECochG thresholds would show more repeatability compared with higher thresholds, and that (iii) thresholds would be lower at lower frequencies, compared to higher frequencies, of the cochlea, as that pattern of hearing loss is typically observed in deaf individuals (26).

## Materials and methods

### Subjects

Fifteen adult CI recipients (mean age 57.8 years; age range 33–75 years, 8 males) with a unilateral CI from Advanced Bionics for >1 year volunteered to participate in the study. All subjects could read and understand English. At the time of recruitment, no subjects had any known neurocognitive impairments likely to impact their ability to participate in the research activities or any known cochlear abnormalities likely to influence their CH measurements. Subjects were recruited from the National Institute of Health Research (NIHR) Nottingham Biomedical Research Center (BRC) Hearing Sciences participant database, the Nottingham Auditory Implant Programme and online advertisements. Written informed consent was obtained from all subjects and the study was approved by the University of Nottingham Faculty of Medicine and Health Sciences Research Ethics Committee and the West Midlands—Black Country Research Ethics Committee.

### Equipment

#### Active insertion monitoring (AIM) system

**Figure 1:**
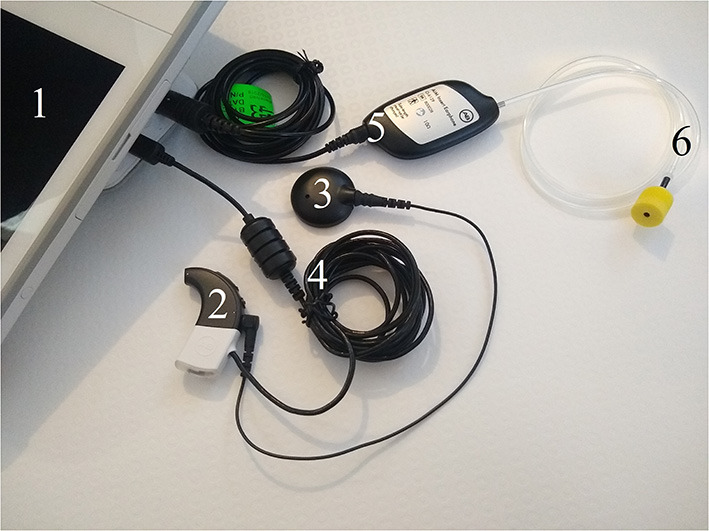
AIM system components. (1) AIM tablet; (2) CI sound processor; (3) CI headpiece magnet and cable; (4) programming cable; (5) AIM insert earphone connector; and (6) acoustic tube with yellow foam ear tip.

The AIM system takes the form of an electronic tablet with pre-programmed CH measurement software “OM Suite” (Advanced Bionics LLC, Santa Clarita, CA) installed, enabling recordings to be made with ease (25). Typically, the AIM system is used by clinicians during CI surgery for real-time CH monitoring. Specifically, intraoperative monitoring during the insertion of the electrode array into the cochlea aims to evaluate any associations between these objective measures of CH and loss of residual acoustic hearing (27, 28). In this project, we repurposed this technology for post-implantation CH self-assessment. All participants received an AIM device, on loan to the University of Nottingham from Advanced Bionics, in order to take their own CH recordings themselves at home. A charger, connection cables, acoustic tubes and foam ear tips were also provided. Each participant also received a separate CI processor and headpiece/magnet so that they did not need to use their own clinical processors to perform the recordings. A photograph of the components of the AIM system is displayed in Figure 1. In addition to the AIM equipment, participants were also sent a memory stick to save data onto and some subjects requested a touchscreen pen. Each AIM system was stored in a carry case also provided by Advanced Bionics.

#### Behavioral assessments

Unaided PTA was performed using a Siemens Unity 2 Diagnostic Audiometer in a soundproof room following the procedure outlined in BSA (29). Participants did not wear their CIs or any other listening devices during this test.

Speech test stimuli were programmed in Python (Python Software Foundation, Beaverton, OR) on a Lenovo Thinkpad laptop and presented in the free field through a Genelec 8030A loudspeaker *via* an external Focusrite sound card. Participants were seated comfortably directly in front of a loudspeaker at a listening distance of 1.5 m with their CIs (and any other contralateral listening devices if usually worn) turned on. Auditory stimuli were calibrated to an average level of 70 dBA, measured at the participant's listening position without the subject present using a sound level meter (Type 2250, Brüel & Kjær, Nærum, Denmark).

Speech test stimuli included 2 Bamford-Kowal-Bench (BKB) sentence lists (30), comprised of 16 sentences in each list, and 3 Arthur Boothroyd (AB) short word lists (31), comprised of 10 words in each list, both presented in quiet and recited by a male speaker. Each BKB list has 50 key words, and each AB short word list has 30 phonemes. In both speech tests, participants were instructed to listen carefully and repeat the sentence/word heard back to the experimenter to the best of their ability. Participants were scored on their ability to correctly identify the pre-determined key words/phonemes.

### CH measurements

The three CH measures were pre-programmed in OM Suite on the AIM tablet. A description of each measurement is provided below. It is important to highlight that the research team used the pre-programmed measurements as pre-set on the AIM devices and did not manipulate the coding of the measurements (beyond selecting some stimulation parameters, which are highlighted as appropriate). Also note that in Advanced Bionics CIs, electrode 1 (e1) is the most apically located electrode and e16 is positioned closest to the base of the cochlea. All measurements were performed on the implanted ear.

#### Electrode impedances

Electrode impedances are recorded by creating a circuit between the intra-cochlear electrode to be measured, and both the ring and case ground electrodes. A biphasic pulse with a current of 32 μA and a phase duration of 18 μs (36 μs in total) is delivered. The voltage on the intra-cochlear contacts with respect to the ground electrodes is recorded using an amplifier inside the implant. This value is digitized and transmitted using the back telemetry function to the sound processor, and then to the software application being used to make the impedance measurement. The voltage is recorded ~7 μs into the current pulse, so it primarily represents the access resistance component of the impedance. The same process is repeated for all 16 of the intra-cochlear electrode contacts and the resulting recorded voltages are used to calculate the impedance, using Ohm's Law. Dividing each recorded voltage by the 32 μA stimulation current provides the impedance value. In this study, impedance data from all 16 electrodes was recorded.

#### eCAPs

Neural Response Imaging is the technique used to record eCAPs *via* the Advanced Bionics CI system (16). In order to record an eCAP threshold, current is delivered between one intra-cochlear electrode contact and the case ground electrode. A biphasic pulse with a phase duration of 32 μs is used for stimulation. Typically, the current used starts at a low level and is increased in steps until a recognizable response is obtained. In this study, the minimum eCAP stimulation level was fixed across the cohort at 100 current units (cu), but the maximum level varied from 250 to 350 cu depending on an individual's comfort level. The recording amplifier is typically configured to record from an electrode contact two positions away from the stimulating contact. This is to avoid the charge and stimulation artifact present at the stimulating contact, yet to record from the same part of the cochlea to which stimulation has been delivered. The recording amplifier is referenced to the ring ground electrode. By separating the case and ring grounds for eCAP measurements, the noise is reduced and the small neural response can be better identified. In order to reject the remaining stimulus artifact, opposite polarity, cathodic leading and anodic leading pulses are used, with the recorded voltages from each being summed. This cancels the artifact but sums the physiological response. Some 64 of these pairs of responses are averaged when recording eCAPs. In this study, eCAP thresholds were only measured at e1, e5, e9 and e13 to gain an insight into auditory nerve function from several regions along the length of the cochlear duct, whilst ensuring the measurement duration was a reasonable length for participants.

#### ECochGs

The ECochG recording capability of the Advanced Bionics CI system records the cochlear microphonic component of ECochG for acoustic stimulation of the cochlea. A burst of acoustic stimulation of 50 ms in duration is delivered *via* an insert earphone. Since CI recipients are typically severe-to-profoundly deaf, the level used for monitoring during insertion of the electrode array is quite high, usually ~100–115 dB SPL. Note that the algorithm used by Advanced Bionics is: Threshold = S – 20 × log10 (C/0.25) where S is the stimulus level in dB SPL and C is the cochlear microphonic amplitude. The software then uses a single stimulus level from which to plot a linear regression. While acoustic stimulation is delivered, the potentials inside the cochlea are recorded from the most apical electrode contact, contact 1. Pairs of acoustic stimuli are delivered with opposite phases. These two recordings are then subtracted to isolate the cochlear microphonic signal. A number of subtracted pairs are averaged, typically 20, after which a data point is plotted on a cochlear microphonic vs. time plot if the signal-to-noise criterion is met (2:1 in this study). In order to estimate the cochlear microphonic amplitude, a fast-Fourier transform is performed on the time domain data, with the bins in the region of the stimulation frequency used to calculate the response amplitude. During clinical use of the AIM system, the intention is to provide real-time feedback to the surgeon, which requires a rapid measurement, producing up to 8 points per second. In this study, EcochG thresholds were recorded at 125, 250, 500, 750, 1,000, 1,500, 2,000, 3,000, and 4,000 Hz using a 115 dB HL tone-burst stimulus.

### Experimental procedure

#### Initial set-up and AIM training

Once each eligible subject had consented and was enrolled onto the study, they were issued an AIM system. Relevant measurement settings were loaded onto each AIM tablet and the performance of the system checked by a researcher prior to being sent to subjects. Once received, participants completed at least one virtual training session with a researcher. They were instructed on how to safely perform their CH measurements and shown how to electronically share data with the research team. Maximum eCAP stimulation levels were also set for each subject during their initial training session. A minimum level of 250 cu was pre-programmed onto the AIM system by the research team and, if necessary, increased to either 300 or 350 cu during the training depending on how comfortable each participant found the stimuli to be. Subjects were also taught how to check certain settings (e.g., ECochG stimulation level and electrodes selected for eCAPs) and advised to perform these checks at the start of each week throughout the study. Note that there were no reported instances of any participant having to change any settings and all data files received were as expected, showing no indication of altered or inconsistent measurement settings. Practice measurement runs were performed during the training session and repeated until both the participant and researcher were confident that all the relevant steps had been learned. Subsequent training sessions were offered on a case-by-case basis as and when required throughout the project.

#### CH measurement period

CH measurements were made by participants themselves at home over a 12-week period, including electrode impedances, eCAPs and ECochGs. Electrode impedances were performed twice a day in order to examine whether recordings remained consistent throughout the day. Subjects were asked to perform the morning (denoted as AM) impedance measurements as close to the start of their day as reasonably possible and to perform the evening (denoted as PM) impedance measurements as close to the end of their day as reasonably possible. eCAPs and ECochGs were performed less frequently in order to minimize participant burden and increase measurement compliance as they were more time consuming for subjects to perform than impedances. eCAPs were performed once a week (i.e., on day 1 of each week) and ECochGs were performed daily in the first week and weekly thereafter (i.e., on days 1–7 during week 1 and then on day 1 of each week from week 2 onwards). Each data collection session lasted ~5–10 min in total, depending on which measurements were performed. AIM systems were returned to the research group after the testing period was completed.

#### Behavioral assessments

In order to investigate the correlation between CI outcomes and CH, subjects were invited to attend an in-person research appointment to complete a hearing test and speech assessments upon completion of their 12-week testing period. Note that behavioral assessments were conducted for descriptive purposes (i.e., to contrast against ECochG data) and not intended for formal data analyses. Due to the global coronavirus (COVID-19) pandemic that was ongoing throughout the study, the research appointment took place soon after the end of the testing period for some participants, whereas for others it was not carried out until several months later. This variation is not deemed remarkable since participants were experienced CI users who were expected to have stable hearing (losses). Importantly, only 13 out of the 15 participants agreed to attend the face-to-face appointment so behavioral data is not available for 2 subjects.

### Data analysis

#### AIM data file conversion

Raw electrode impedance, eCAP and ECochG data files were converted from JSON to Microsoft Excel files using conversion software provided by Advanced Bionics. Data from Excel files were extracted using MATLAB (Mathworks, Natick, MA) and analyses were carried out in IBM SPSS Statistics for Windows Version 28.0 software (IBM Corp., Armonk, New York).

#### Electrode impedances

Scatter graphs of each individual's impedance measurements for all electrodes were generated, and standard deviations calculated to assess variation. In order to examine whether impedances differed between morning and evening recordings, and between different cochlear regions, eight electrode impedance values were generated per subject and entered in a repeated measures analysis of variance (RM- ANOVA). Specifically, mean individual impedance values (from the entire dataset) for e1-e4, e5-e8, e9-e12, and e13-e16 were calculated for AM and PM separately. The first within-subject factor was “timing” which had two levels (AM and PM) and the second was “cochlear region” which had four levels (e1-e4, e5-e8, e9-e12, and e13-e16).

#### eCAPs

Individual eCAP thresholds were plotted for the entire testing period in order to visually assess the data and identify potential cochlear dead regions. Measurement variation at each recording electrode was assessed using boxplots. Importantly, cochlear dead regions refer to parts of the cochlea where auditory neurons and/or inner hear cells are damaged or dysfunctional (32). Assessing cochlear dead regions is of great scientific and clinical importance as they inform hearing device programming and decisions (32, 33), and influence clinical outcomes (32, 34). Furthermore, changes in cochlear dead regions following administration of hearing loss treatments which aim to restore hair cell and/or auditory neuron function (e.g., 7) could help assess the success of such solutions.

#### ECochGs

Average thresholds were calculated for each individual at each frequency at which a successful threshold was derived. The relationship between measurement consistency and ECochG threshold was assessed with a correlation and boxplots were produced to investigate the spread of ECochG thresholds at each frequency across the group.

#### Behavioral assessments

A series of correlations were performed between AB phoneme scores vs. impedances and eCAPs to investigate the relationship between CI outcome vs. impedance variability and potential dead regions, respectively. A single standard deviation value was calculated from all impedances (across the entire electrode array and across all 12 weeks) for each individual and correlated against AB scores. Four mean eCAP thresholds per subject, one for each recording electrode (e1, e5, e9, and e13) were also calculated (absent thresholds were excluded), as well as the total number of successful thresholds (out of a maximum of 48 per subject). These values were then entered into five correlations with corrections made for multiple comparisons as appropriate.

## Results

### Participant compliance

Participant compliance was exceptionally high. A total of 27 impedances were missing out of a possible 2,520 recordings across the cohort. Specifically, compliance with twice-daily impedance recordings was either 99 or 100% in 10 subjects, and did not drop below 96% across all 15 participants in the study. Only 1 ECochG recording was missed from a possible 270 across the entire group and no eCAP measurements were missed by any participant. Group level compliance with recordings was 98.9% for impedances, 100% for eCAPs and 99.6% for ECochGs.

### Data exclusion

Electrode impedances from e16 in subject 1 and e14 in subject 14 were excluded from data analyses. These anomalous results were removed as they were consistently abnormally high (65 kΩ in subject 1 and ~30 kΩ in subject 14) throughout the testing period, indicating that the electrode contacts in question may be sitting in an extracochlear location and/or switched off.

### CH measurements

#### Electrode impedances

**Figure 2:**
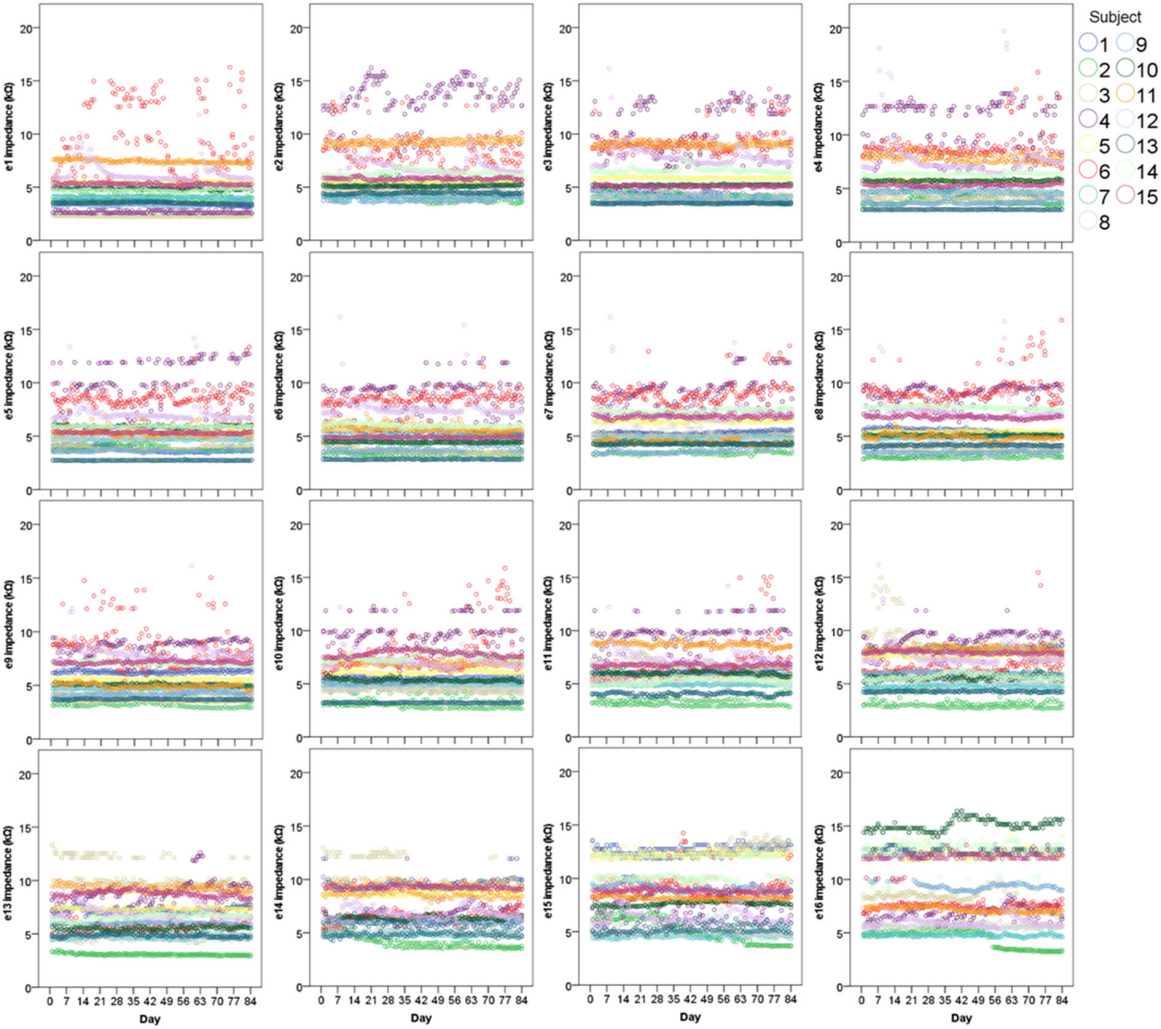
Scatter graphs displaying individual electrode impedances at all 16 electrodes over 12 weeks (*N* = 15). Two points (both AM and PM) are plotted for each day.

Scatter graphs showing individual measurements over time are displayed in Figure 2. As hypothesized, impedances remained very stable over time in the majority of cases across all 16 electrodes. Importantly, even the most varied data still generally fell within the accepted normal clinical range of 2–15 kΩ (T Nauwelaers 2022, personal communication, 17 Oct).

**Figure 3:**
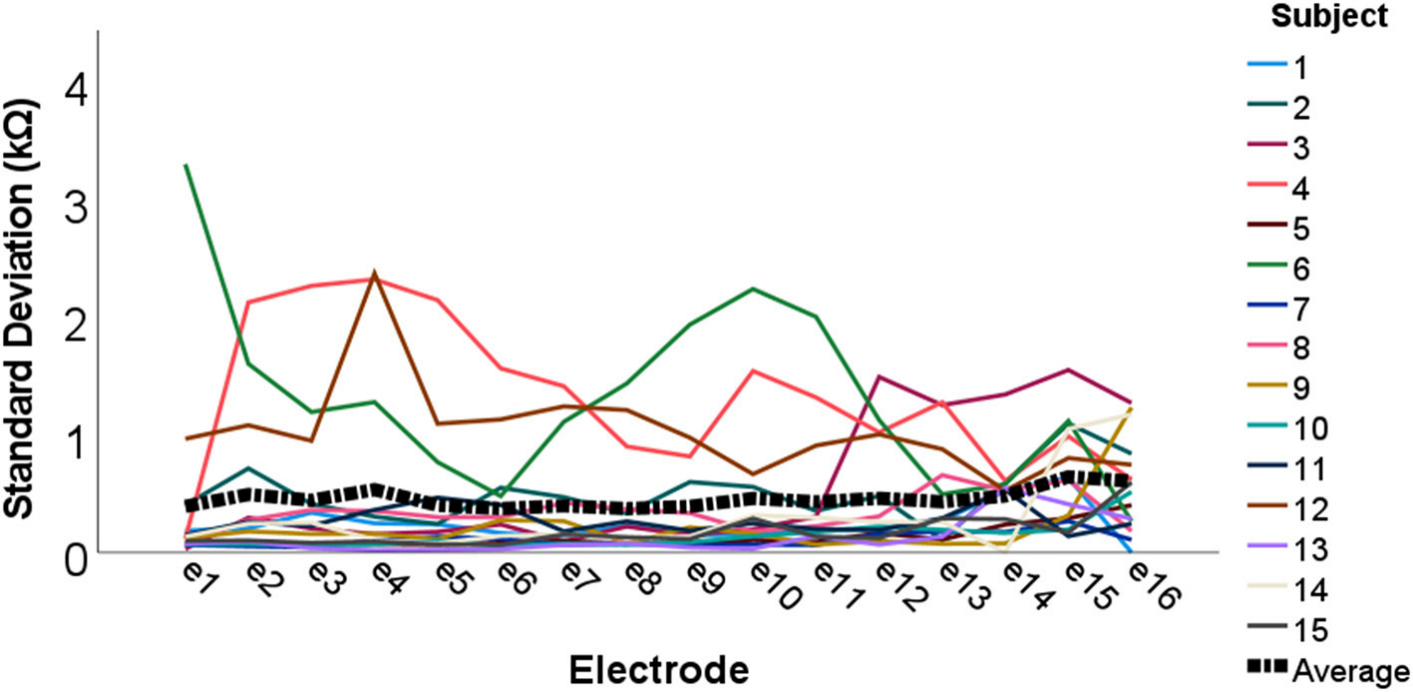
Individual and group level electrode impedance standard deviations plotted across the electrode array. Colored lines represent each individual participant (*N* = 15), and the black dotted line shows group average standard deviations.

**Figure 4:**
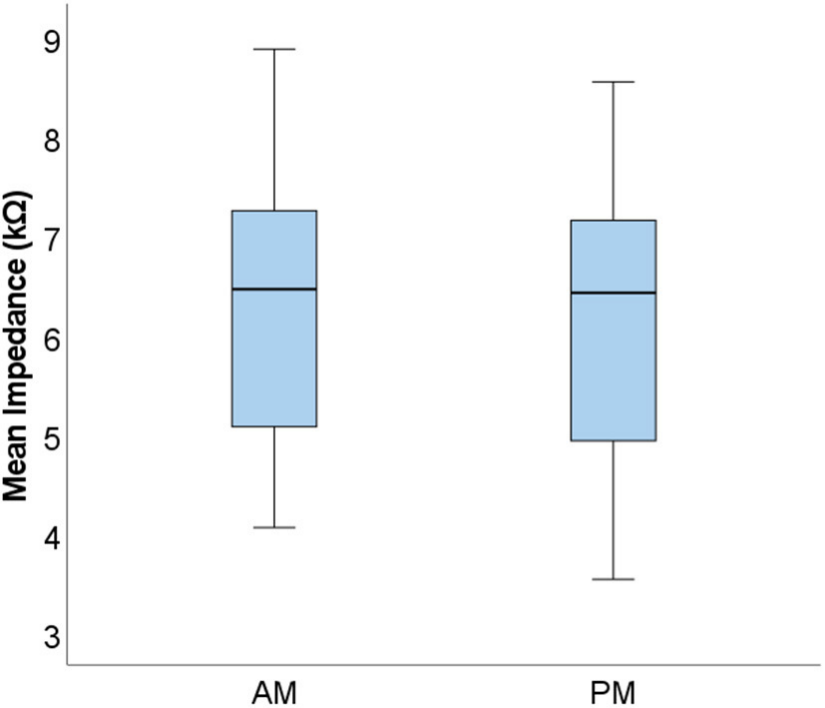
Boxplots displaying group level mean AM and PM impedances. Mean values calculated from the four electrode groupings are shown (*N* = 15).

To investigate measurement stability across the electrode array, standard deviations were calculated and plotted for each electrode contact, for each subject (see Figure 3). Variation was minimal across the electrode array for the majority of participants, with only four cases in which the standard deviation appeared noticeably higher, though all these measurements still fell within the clinically accepted range (T Nauwelaers 2022, personal communication, 17 Oct). Mean group level standard deviations at each electrode suggested highly stable impedances across the subject group, with a slight increase in variation at either end of the array, particularly toward the basal end of the cochlea (e15 and e16). An RM-ANOVA was performed to investigate morning vs. evening and cochlear region (e1-e4, e5-e8, e9-e12, and e13-e16) differences. There was a statistically significant main effect of timing [*F*_(1, 14)_ = 5.808, *p* = 0.030] and cochlear region (F(1.316, 18.419) = 9.565, *p* =0.004), however there was no significant interaction between the two [*F*_(3, 42)_ = 0.864, *p* = 0.467]. On average, impedances were 0.111 kΩ higher (*p* = 0.030) in AM compared to PM recordings (see Figure 4). Pairwise comparisons with Bonferroni-adjusted alpha levels revealed that impedances were the highest in the most basal electrode grouping (e13-e16). Specifically, they were 2.182 kΩ (*p* = 0.045), 2.236 kΩ (*p* = 0.025) and 1.688 kΩ (*p* = 0.026) higher than the e1-e4, e5-e8, and e9-12 groupings, respectively. No other pairwise comparisons were statistically significantly different.

#### eCAPs

**Figure 5:**
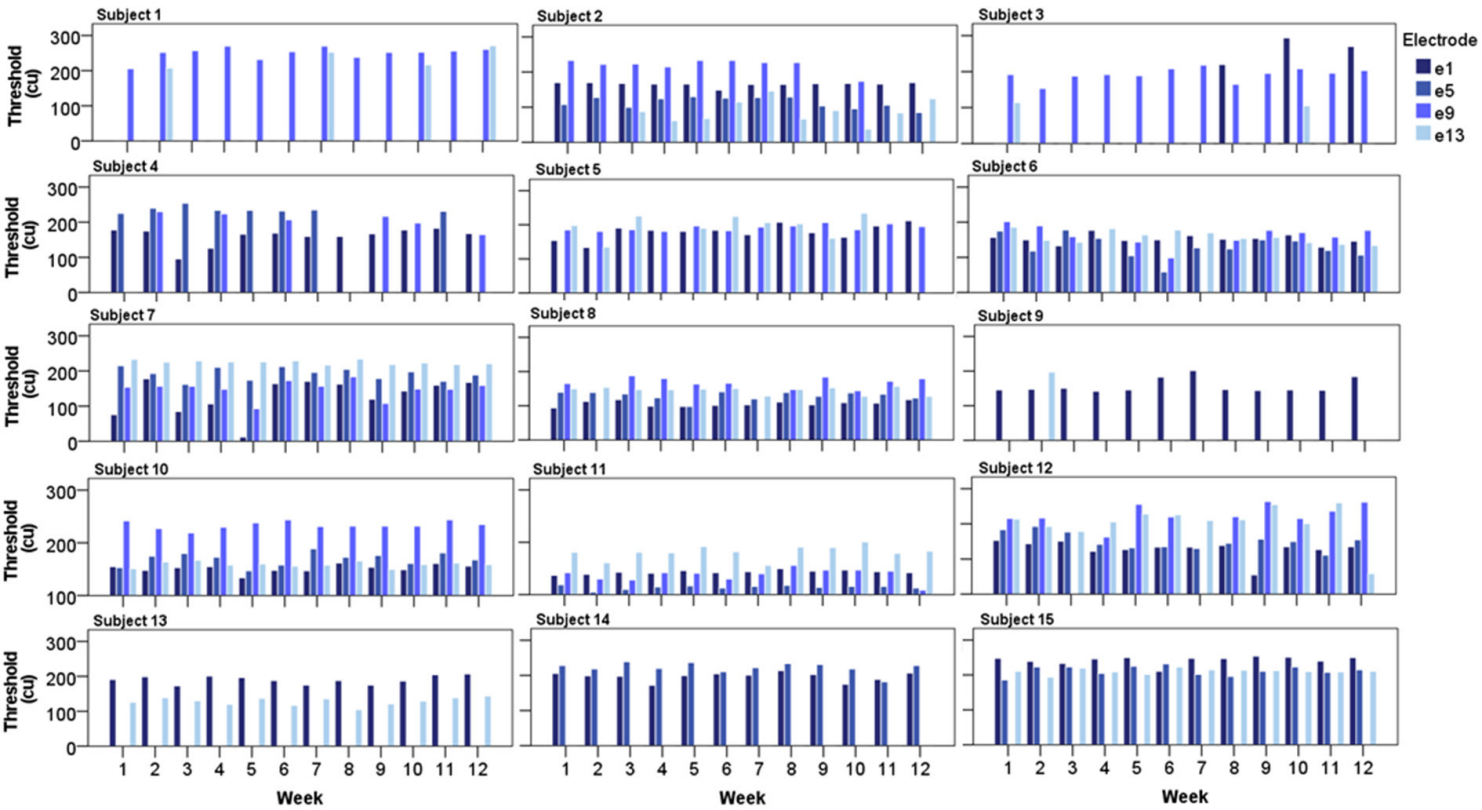
Bar charts displaying each subject's eCAP threshold from all four recording electrodes over the 12-week testing period (*N* = 15).

Individual eCAP thresholds across the testing period are displayed in Figure 5. In all subjects, a successful eCAP threshold was consistently derived every week from a minimum of one electrode. In the majority of cases, each subject's thresholds appeared stable and highly repeatable over time, as hypothesized. There appeared to be greater variation in responses where thresholds were higher compared to lower thresholds, which seemed to be more consistent over time. In some cases, a threshold was consistently absent over all 12 weeks at a particular electrode, as indicated by missing bars in Figure 5, which was suggestive of potential cochlear dead region(s) in the subjects in question.

**Figure 6:**
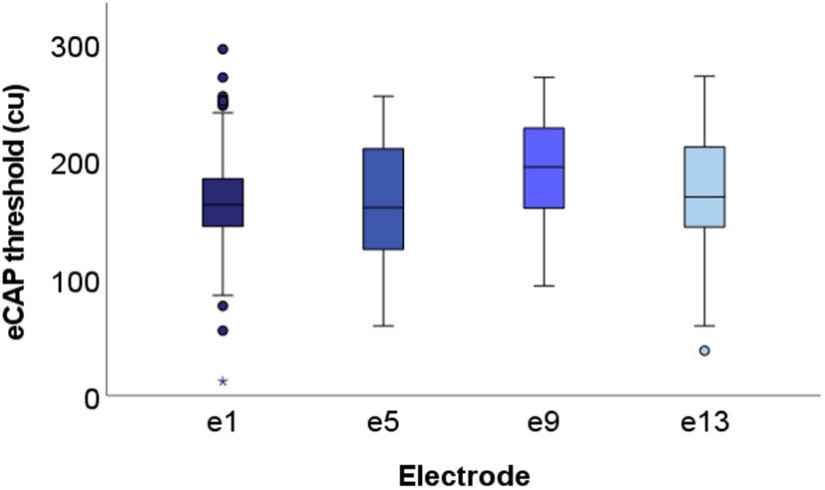
Boxplots displaying group level eCAP data at each of the four recording electrodes. All successful thresholds from the entire cohort (*N* = 15) are included. Specifically, 159, 116, 117, and 122 thresholds (out of a maximum of 180) are included from e1, e5, e9, and e13, respectively. Median values fell within the range of 150–200 cu across all four electrodes.

The spread of the data across at each recording electrode is visualized in boxplots displayed in Figure 6 (only successful thresholds are included). Although the greatest number of outliers are found in thresholds derived from e1, these data also have the tightest interquartile range. Conversely, eCAPs from e5 are the most spread out. Furthermore, eCAP thresholds did not significantly change from the base (e13) to the apex (e1) of the cochlea.

#### ECochGs

Table 1 summarizes each subject's ECochG thresholds. The number of valid thresholds varied considerably, and, in most cases, were low, though high consistency was observed in a handful of subjects. The number of hearing frequencies the thresholds were recorded at also varied extensively from subject to subject, with some individuals only showing a successful response at 2 of the frequencies recorded at, and others at all 9. In order to assess whether measurement consistency (i.e., the number of valid thresholds) was higher when the ECochG thresholds were lower, a correlation was performed between valid and average thresholds. As hypothesized, a statistically significant negative correlation was observed (τb = −0.160, *n* = 75, *p* = 0.030), indicating that lower (i.e., better) ECochG thresholds showed more repeatability over time compared with higher thresholds.

**Table 1:** Summary of each subject's ECochG threshold (*N* = 15).

		**Frequency (Hz)**								
**Subject**	**Threshold**	**125**	**250**	**500**	**750**	**1,000**	**1,500**	**2,000**	**3,000**	**4,000**
1	Valid (%) Average (dB HL)				6 94			11 85	28 85	
2	Valid (%) Average (dB HL)	72 70	100 80	100 82	100 89	100 98	72 96	100 96	17 99	83 96
3	Valid (%) Average (dB HL)		17 88	89 96	100 96	100 96	100 94	100 93	33 97	100 90
4	Valid (%) Average (dB HL)				11 101	6 104	6 97	11 98	28 98	89 96
5	Valid (%) Average (dB HL)				6 93					6 88
6	Valid (%) Average (dB HL)			28 99		33 100	11 100	6 102		17 98
7	Valid (%) Average (dB HL)			6 92	22 91	17 92		6 87		44 86
8	Valid (%) Average (dB HL)	100 55	100 61	100 86	78 96	100 85	100 86	100 92	100 95	100 82
9	Valid (%) Average (dB HL)							11 100	33 98	100 94
10	Valid (%) Average (dB HL)					6 94	6 87	17 85		61 86
11	Valid (%) Average (dB HL)		44 90	89 94	89 97	83 95	83 91	89 91	89 93	89 91
12	Valid (%) Average (dB HL)		6 85	6 92	28 90		22 86	6 85	89 81	100 80
13	Valid (%) Average (dB HL)				6 97					11 87
14	Valid (%) Average (dB HL)									11 84
15	Valid (%) Average (dB HL)		1 86				1 91			3 86

A validity score of 100% at a given frequency indicates that a successful threshold was derived during all 18 ECochG tests performed throughout the testing period. Validity scores vary considerably from person to person and from frequency to frequency. As expected in a group of CI recipients, hearing thresholds are high.

**Figure 7:**
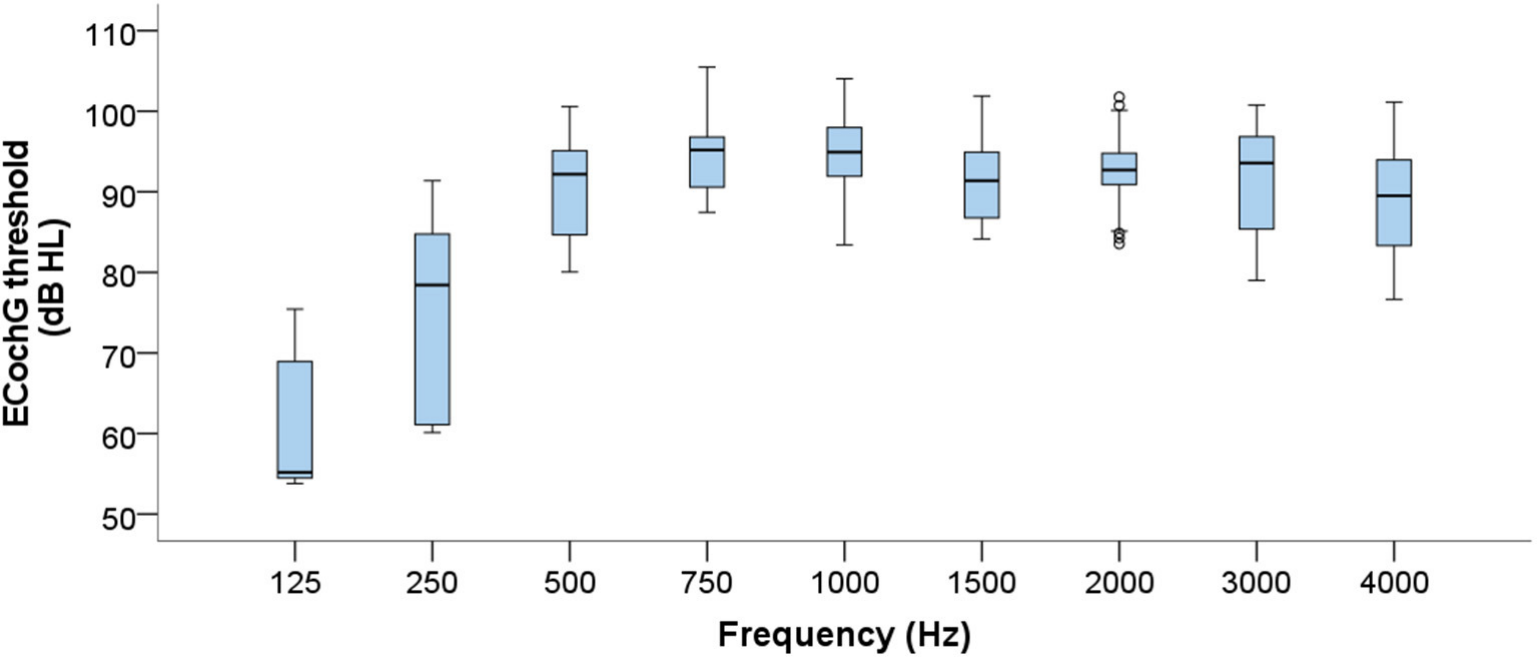
Boxplots displaying group level ECochG thresholds across the frequency range. Data from all 15 subjects are compiled, resulting in considerable differences in numbers of valid thresholds at each frequency.

Figure 7 illustrates the spread of ECochG thresholds at each frequency across the subject group. The two lowest frequencies display the greatest variation, whereas the mid-frequencies (750–2,000 Hz) are the most consistent. As expected, ECochG thresholds overall worsen from 125 to 750 Hz after which they generally fall within the profound hearing loss range. However, since the overall number of successful thresholds recorded in the group differed quite considerably at each frequency, the ECochG data must be interpreted with this caveat in mind.

### Behavioral assessments

BKB sentence test and AB short word mean test scores from the 13 subjects who completed behavioral assessments are displayed in Table 2. Most participants demonstrated high levels of sentence recognition, as indicated by the high BKB scores. As expected, AB phoneme identification scores were lower in almost all cases since due to a lack of contextual information, making the test more challenging (35).

**Table 2:** Mean speech test scores (*N* = 13).

**Subject**	**BKB score (% correct)**	**AB score (% correct)**
1	45.6	50.0
3	90.4	74.4
4	73.2	46.7
5	93.8	77.8
6	92.7	77.8
7	65.4	41.1
9	97.9	67.8
10	93.2	77.8
11	99.0	83.3
12	95.6	85.6
13	89.6	54.4
14	70.3	66.7
15	81.0	74.4

Subjects scored highly on the BKB sentence test, with 12 of 13 subjects attaining at least 70% correct. Results from the AB short word test were lower, but many participants still performed well.

The relationship between AB phoneme scores and cochlear health was assessed using a series of correlations. Since ECochGs were, as anticipated, highly erratic, those thresholds were not included in the analyses. Furthermore, BKB scores were not correlated due to ceiling effects since most subjects performed well.

In order to assess whether subjects with the highest variability in their impedance measurements had worse speech perception compared with those with lower variation, overall impedance standard deviations per individual were correlated against AB phoneme scores. No statistically significant correlation was observed (*r* = 0.138, *n* = 13, *p* = 0.653).

To examine the relationship between potential cochlear dead regions and speech performance, four correlations between AB scores and mean eCAP thresholds for each electrode were performed. Weak correlations were observed in all cases except between AB scores and eCAPs derived from e5, where a moderate negative correlation was found. However, none of the four correlations between speech scores and e1 thresholds (*r* = 0.092, *n* = 12, *p* = 0.776), e5 thresholds (*r* = −0.666, *n* = 8, *p* = 0.071), e9 thresholds (τb = −0.087, *n* = 9, *p* = 0.750) or e13 thresholds (τb = −0.132, *n* = 11, *p* = 0.580) were found to be statistically significant. An additional correlation of the total number of successful eCAP thresholds vs. AB scores was also carried out but no statistically significant correlation was observed once again (*r* = 0.352, *n* = 13, *p* = 0.238).

PTA air conduction thresholds were derived from the implanted ear at the four key speech frequencies (500, 1,000, 2,000, and 4,000 Hz). From the 13 subjects tested, only 4 thresholds in total were successfully recorded, both at the two lower frequencies. Specifically, subject 6 had thresholds of 105 and 120 dB HL and subject 9 had thresholds of 75 and 85 dB HL, at 500 Hz and 1,000 Hz, respectively. In all other instances, no response was recorded at a maximum stimulation level of 115 dB HL at 500, 2,000, and 4,000 Hz and 120 dB HL at 1,000 Hz.

It was initially hypothesized that ECochGs would only be recorded in subjects with some degree of residual hearing, measured using PTA. However, a valid ECochG threshold was recorded at least once in every subject, with some participants demonstrating very high levels of ECochG threshold consistency but no PTA responses. Furthermore, ECochG results were not closely linked to residual hearing ability in the two individuals who did have PTA hearing thresholds. Specifically, subject 6 only showed valid ECochG thresholds at 500 and 1,000 Hz at approximately one third of the attempted measurements and subject 9 did not have any valid ECochG thresholds at the same frequencies over the entire testing period.

## Discussion

To our knowledge, this is the first study to have CI recipients self-assess their CH without any clinician input on a daily basis over a 12-week period. The purpose of our work was 2 fold: (i) to evaluate the feasibility of using a CI to measure CH through participant self-assessment and (ii) to examine the pattern of electrode impedances, eCAPs and ECochGs over time. Not only were subjects very highly engaged with taking daily recordings, achieving excellent compliance results across all three measurements of interest, the results of the recordings themselves were as one would expect in the clinic, even with participants performing them at home, independently and unsupervised.

Electrode impedances were comparable with those collected from a considerably larger group of Advanced Bionics recipients in a recent study (6). In the vast majority of cases, impedances remained highly stable over time. Importantly, even when some degree of variation was observed, albeit minimal, values still fell well within the accepted normal clinical range (T Nauwelaers 2022, personal communication, 17 Oct). In the two instances (of excluded data) when values fell far beyond the normal range, results were consistently high throughout the whole testing period. This suggests that factors relating to the CI itself, such as extracochlear electrode contacts, for example, triggered the high impedances as opposed to variation in the measurements themselves (6, 36).

Interestingly, we found that impedances recorded in the morning were higher than those performed in the evening. This may have been due to the fact that AM impedances were typically performed after a period of no electrical stimulation (i.e., overnight sleep without CI use), and previous studies have shown a reduction in impedance values following electrode stimulation, although this effect is typically observed in early days post-implantation (37, 38). It is plausible to assume impendences then steadily decreased throughout the day as a consequence of CI use, particularly since the difference itself was incredibly small (0.111 kΩ). Furthermore, consistent with previous studies (6, 39), we also found that impedances were the highest toward the base of the cochlea, at the site of surgery. This is likely due to increased osteogenesis, fibrous tissue and scarring at these cochlear regions (40).

eCAP measurements were also, in the vast majority of cases, stable over time, with every subject showing 100% measurement consistency at, at least one electrode. When eCAPs were recorded intermittently, this was typically associated with higher thresholds. It is plausible that in these instances, the maximum stimulation level and threshold were close together, resulting in erratic recordings and causing variation. A limitation of our study is that maximum stimulation levels were capped between 250 and 350 cu. Therefore, these higher thresholds might only have been reached on some occasions and not on others as a result of insufficient stimulation of the nerve (17, 18). It is likely that increasing the stimulation level in these cases would have resulted in more consistently successful thresholds being derived. However, due to participant comfort concerns and the self-assessment nature of the study, we opted not to further increase maximum eCAP stimulation levels beyond 350 cu, even when patients felt they could comfortably tolerate higher sound levels.

Just over half of all subjects recorded no eCAPs at all from particular electrodes, potentially suggesting poorer spiral ganglion coverage in corresponding regions of the cochlea (22, 23). Though the lack of eCAPs cannot guarantee the existence of dead regions, particularly since it is likely that higher stimulation levels, without the maximum limit of 350 cu, would have resulted in additional thresholds being revealed, it is still reassuring that all electrodes had consistently normal impedances. Our eCAP data suggest that daily eCAP measurements could, possibly, form one appropriate way of measuring the effectiveness of novel treatments for hearing loss. Specifically, if a therapy can be shown to reduce eCAP thresholds or enable eCAPs to be recorded from electrodes not previously possible, that could suggest the success of the therapy in increasing the population of local spiral ganglion neurons. However, an important limitation of our work is that that we only recorded from four electrodes across the array which significantly reduced the temporal resolution of our findings. Future work could implement the use of other methods that have been described to more intensively assess cochlear dead regions, such as panoramic eCAPs, for example (41, 42).

Surprisingly, although the participants were all profoundly deaf CI recipients with little-to-no expected residual hair cell function, a successful ECochG was measured at least once from at least one frequency in every individual. However, the thresholds were highly inconsistent, both between and within subjects, with only four participants displaying good levels of measurement consistency. As expected, significantly more successful recordings were made when thresholds were lower (i.e., better), with very few thresholds beyond 100 dB HL measured (though this was expected given the 115 dB HL stimulation level). Furthermore, since the ECochGs in this study were limited by their reliance on good acoustic tube and foam tip positioning, it is likely that poor placement by the subject would have impacted the recordings, which is another factor likely to have contributed to the variance. Interestingly, successful ECochGs were most likely to be recorded at 4,000 Hz, with approximately double the number of successful thresholds at this frequency compared to most other frequencies, though no behavioral thresholds were recorded at 4,000 Hz when subjects performed PTA. Although some studies have shown a strong correlation between ECochG thresholds and audiometric thresholds (11), we found no such relationship. In fact, we recorded many more ECochGs than audiometric thresholds, even though typically, ECochG signals are found at or above hearing thresholds (43). These contrasting findings highlight the unpredictable nature of the ECochG recording, particularly if it is measured by participants themselves as, unlike electrode impedances and eCAPs, the ECochG relies on an external acoustic stimulus.

We also investigated whether individuals with the highest variability in their impedance measurements, or ones with potential cochlear dead regions, differed in terms of their CI outcomes compared to subjects with lower impedance variation or those without absent eCAPs. However, we found no relationship between our CH measures and AB speech scores. This may be due to ceiling effects since the cohort were generally good CI performers and scored highly in the speech tests. This self-selection factor is a common limitation in this field since it tends to be those individuals who are doing well with their implants that come forward to participate in research. In addition, the variation observed in impedances across the group was only minimal and recordings fell within normal limits anyway (T Nauwelaers 2022, personal communication, 17 Oct). Perhaps if greater variation was observed with a higher number of very high or very low impedances, a stronger relationship with behavioral measures may have been revealed. In addition, it is possible that our small sample did not have the power to reveal the extent of the relationship between CI outcomes and CH, if any, particularly since many of the eCAP correlations were run with only a handful of subjects as not every individual had an eCAP at every electrode (and behavioral speech data was not available for every participant).

To summarize, it is feasible for CI users to record cochlear heath data using their CIs, thus illustrating the power of using a CI to intensively assess CH. Exceptionally high participant compliance levels further indicate that subjects can themselves successfully monitor CH, even with an intensive data collection schedule of twice a day for 12 weeks. Electrode impedances and eCAPs, in particular, show good measurement consistency, making them worthy of further consideration and investigation when developing tools to objectively evaluate CH in early-phase trials of adjunctive cell-based therapies. Future work should investigate CH changes immediately following implantation in new CI recipients to assess early patterns post-implantation. Further studies involving a greater number of subjects with a greater degree of variation in CI outcomes and speech performance are required to determine the use of CH measures for assessing variation in CI outcomes.

## Data availability statement

The raw data supporting the conclusions of this article will be made available by the authors, without undue reservation.

## Ethics statement

The studies involving human participants were reviewed and approved by University of Nottingham Faculty of Medicine and Health Sciences Research Ethics Committee and the West Midlands - Black Country Research Ethics Committee. The patients/participants provided their written informed consent to participate in this study.

## Author contributions

FM, AS, PB, TN, and DH were involved in the conception and design of the work. FM recruited participants and collected the data. Data analysis and interpretation was primarily conducted by FM, with guidance and advice offered by AS, PB, and DH. FM wrote the manuscript with technical input from PB. AS and DH commented on and reviewed the manuscript. All authors contributed to the article and approved the submitted version.

## Funding

This work was joint funded by Rinri Therapeutics and a UKRI Innovation Scholars secondments: biomedical sciences award (awarded to FM, project number 70387). This paper presents independent research supported by the National Institute for Health Research (NIHR). The funders were not involved in the study design, collection, analysis, interpretation of data, the writing of this article, or the decision to submit it for publication.

## Conflict of interest

Author FM was seconded to Rinri Therapeutics Ltd as part of their Innovate UK award. Author PB was employed by Advanced Bionics GmbH. The remaining authors declare that the research was conducted in the absence of any commercial or financial relationships that could be construed as a potential conflict of interest.

## Publisher's note

All claims expressed in this article are solely those of the authors and do not necessarily represent those of their affiliated organizations, or those of the publisher, the editors and the reviewers. Any product that may be evaluated in this article, or claim that may be made by its manufacturer, is not guaranteed or endorsed by the publisher.

## Author disclaimer

The views expressed in this article are those of the author(s) and not necessarily those of the NHS, the NIHR, or the Department of Health and Social Care.
